# Re-assessing the role of the fecalith in acute appendicitis in adults: case report, case series and literature review

**DOI:** 10.1093/jscr/rjaa543

**Published:** 2021-01-29

**Authors:** Nguyen Tran, Valerie-Sue Emuakhagbon, Bradford T Baker, Sergio Huerta

**Affiliations:** University of Texas Southwestern Medical School, Dallas, TX, USA; Department of Surgery, VA North Texas Health Care System, Dallas, Texas, USA; Department of Pathology, VA North Texas Health Care System, Dallas, TX, USA; University of Texas Southwestern Medical School, Dallas, TX, USA; Department of Surgery, VA North Texas Health Care System, Dallas, Texas, USA

## Abstract

Appendicitis in adults is thought to occur because of luminal obstruction from a fecalith. We present a unique case of a patient who had her entire appendiceal lumen occupied by a fecalith (5.0 cm long) but had no appendicitis. We reviewed the records of 257 veterans who underwent surgical intervention at our institution for the management of acute appendicitis. Fecaliths occurred in 15.6% of patients. At laparotomy, 20.6% had a perforated appendix; pathology showed fecaliths in 20.8% of specimens. A review of the literature inclusive of 25 series showed fecaliths in 33.3% of patients with a normal appendix, 23.5% of patients with acute appendicitis and 24.9% with perforated appendicitis. These data show that appendicitis is not a common cause of fecalith obstruction in adults.

## INTRODUCTION

Conventional teaching in the pathophysiology of acute appendicitis in adults dictates luminal obstruction from a fecalith (or less likely from foreign bodies, parasites, and tumors), which leads to stasis, bacterial overgrowth, inflammation, ischemia and perforation [1]. However, this classic concept, first proposed by Fitz and Mattestock [2], has been questioned by investigators demonstrating a wide range of fecaliths in patients with appendicitis (1.4–67.0%) [3].

Further, intraoperative management of appendiceal pressure in patient with appendicitis revealed that the inflammatory process might be the culprit of luminal obstruction rather than obstruction leading to inflammation [3]. Thus, there is a competing theory that obstruction is the result of inflammation and not the other way around [4].

The classical teaching of a fecalith leading to appendicitis has been challenged by several investigators [4–6], but it has not been systematically analyzed. Current strategies in the management of acute appendicitis include non-operative management (NOM), but fecaliths have been shown to be a predictor of failure to NOM [7]. Thus, a re-assessment of its role in acute appendicitis is timely.

**Figure 1:**
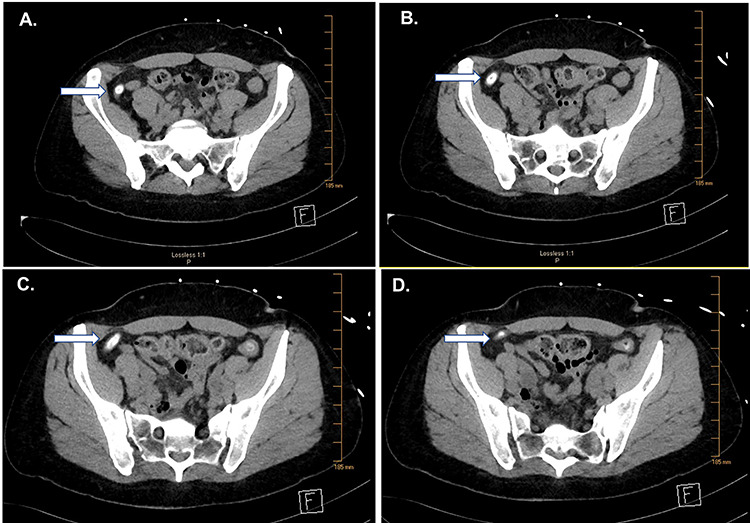
CT of the abdomen and pelvis. Serial images (**A**–**D**) from cephalad to caudal show a large fecalith (arrows).

In the present report, we present a unique patient with a very large fecalith occupying the entirety of the appendiceal lumen; we reviewed all appendectomies in our institution for the past 12 years to assess the role of fecaliths in appendicitis. We then reviewed the literature.

## CASE REPORT

A 50-year-old veteran woman presented to the emergency department (ED) with a 1-day history of abdominal pain, nausea and multiple episodes of vomiting. Her pain was intermittent, severe and poorly localized to the right lower quadrant. She had had similar episodes necessitating ED visits in the past several months, and pain medication had been prescribed, which improved her symptoms. Her past medical history is significant for inflammatory bowel disease, gastroesophageal reflux disease, post-traumatic stress disorder and fibromyalgia. She is a current smoker (4–5 cigarettes a day) but denies alcohol consumption or intravenous drug use. Her medications include ondansetron 8 mg by mouth three times a day, sertraline 50 mg by mouth twice a day and tramadol 50 mg per mouth as needed for pain.

On physical examination, her temperature was 97.9°F, her heart rate was 70 beats per minute, blood pressure 148/88 mmHg, respiration rate of 20 breaths per minute, her body mass index was 28.6 kg/m^2^. She was alert and oriented, but in clear distress from abdominal discomfort, rocking back and forth in bed and moaning from this pain. Her heart and lungs were clear. Her abdominal examination demonstrated no abdominal distension and no rigidity. She was tender to palpation on the right lower quadrant.

Serology analysis showed a white blood cell count of 7.7 K/μL (normal = 4–11 K/μL), Hemoglobin of 12.7 g/dL (normal = 11.5–15.3), hematocrit of 38.5% (normal = 34–45%) and platelets of 334 K/μL (normal = 140–400 K/μL). Electrolytes were all normal, and creatinine was 0.91 mg/dL (normal = 0.50–1.10 mg/dL). Lactic acid levels were 2.1 mmol/L (normal = 0.5–2.2 mmol/L). Her modified Alvarado score was four.

Computed tomography (CT) imaging showed an appendix with an appendicolith with mild dilation (9 mm) and no peri-appendiceal inflammatory changes (Fig. 1). Compared to a CT scan obtained in 2013, this study showed an appendicolith with mild dilation.

**Figure 2:**
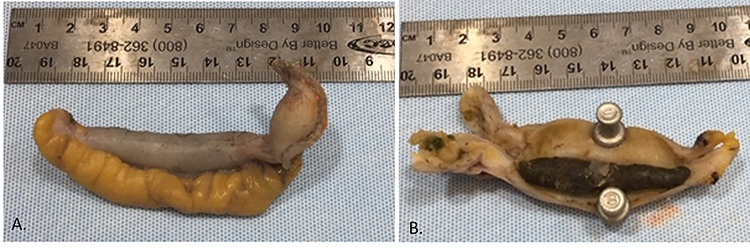
Appendiceal specimen received by pathology (**A**). There is a 5.0 × 0.8-cm tan/dark green fecalith filled within the lumen of the appendix (**B**).

Given these findings, she was taken to the operating room for a laparoscopic appendectomy. Exploratory laparoscopy demonstrated no evidence of acute or chronic appendicitis. Pathological examination of the appendix demonstrated an appendix entirely occupied by a fecalith and no evidence of appendicitis (Fig. 2).

## CASE SERIES

Of 257 patients who were operated on for acute appendicitis at the VA North Texas Health Care System from 2005 to 2017 only 40 had fecaliths (15.6%) [Renteria and Huerta, 2018]. Of 257 patients who underwent an appendectomy initially for acute appendicitis, 38 (14.8%) had gangrenous appendicitis. Of patients with gangrenous appendicitis seven (18.4%) had an appendicolith. Fifty-three (20.6%) had a perforated appendix despite carrying an initial diagnosis of acute appendicitis. Of patients with perforated appendicitis, 11 (20.8%) had a fecalith.

## REVIEW OF THE LITERATURE

Between 1939 and 2019 a total of 25 series, inclusive of 94,592 patients, were reviewed. The average age of patients was reported in 11 studies (38.2 years old ±13.3 standard deviation [SD]). The overall fecalith in appendiceal specimens was 20.7% (range 1.4–67.0%). Fecaliths occurred with a frequency of 20.3% (range 5.3–100%) in 11 reports documenting a normal appendix. Fifteen studies reported an average of 23.5% (range 1.5–65.1%) fecaliths in patients with acute appendicitis. In patients with perforated appendicitis, fecaliths were reported in 24.9% (range 10.2–39.1%) of 10 reported series (Table 1).

**Table 1:** Frequency of fecaliths in the appendix, overall, a normal appendix, acute appendicitis and perforated appendicitis

Year	Number of patients	Average age	Overall fecalith (%)	Fecalith in normal appendix (%)	Fecalith in acute appendicitis (%)	Fecalith in perforated appendicitis (%)	Reference (PMID)
1939	372	NR	67.0	NR	NR	NR	Bowers *et al.* [12]
1943	91	NR	33.0	NR	NR	NR	Steinert *et al.* [13]
1963	71000	NR	44.3	NR	NR	NR	Collins *et al.* [14]
1965	240	NR	33.0	NR	NR	NR	Shaw *et al.* [15]
1979	55	NR	NR	40.0	NR	NR	Burkitt *et al.* [16]
1981	276	NR	16.7	13.0	45.7	39.1	Butler *et al.* [17]
1981	3003	NR	3.6	5.3	6.3	NR	Chang *et al.* [18]
1985	13	25	23.0	NR	NR	NR	Jones *et al.* [9]
1985	63	23	52.0	NR	NR	NR	Jones *et al.* [9]
1990	101	NR	NR	26.7	NR	NR	Andreou *et al.* [19]
1990	3124	NR	3.2	NR	NR	NR	Andreou *et al.* [19]
1990	405	NR	6.3	NR	NR	NR	Babekir *et al.* [20]
2006	200	NR	54.0	NR	26.0	28.0	Herscu *et al.* [21]
2006	2660	NR	3.7	6.3	3.5	NR	Marudanayagam *et al.* [22]
2008	427	24.4	1.4	NR	NR	NR	Sgourakis *et al.* [23]
2010	518	30.9	1.5	NR	1.5	NR	Makaju *et al.* [24]
2012	723	29.3	36.1	44.8	7.3	11.5	Engin *et al.* [25]
2012	4670	NR	3.6	60.5	39.5	NR	Chandrasegaram *et al.* [26]
2013	722	NR	13.7	31.6	12.0	27.5	Singh *et al.* [4]
2014	1357	32	13.7	34.6	65.1	10.2	Ramdass *et al.* [27]
2014	339	40.8	23.3	NR	63.2	36.7	Kim *et al.* [28]
2015	150	NR	13.3	NR	11.8	17.5	Iqbal *et al.* [29]
2017	225	69.4	5.8	NR	10.4	NR	Khan *et al.* [5]
2017	111	38	NR	100.0	NR	NR	Khan *et al.* [5]
2018	257	45.4	15.6	NR	NR	20.6	Renteria *et al.* [8]
2019	1007	38.4	11.1	NR	8.9	21.7	Westfall *et al.* [30]
2019	445	54.5	NR	NR	18.0	36.0	Kulvatunyou *et al.* [31]
2019	2038	44.9	8.0	6.8	33.0	NR	Moskowitz *et al.* [32]
Total	94592	38.2 ± 13.3^a^	20.3	33.6	23.5	24.9	

^a^Average age ± SD. NR, not reported.

## DISCUSSION

A fecalith (also known as stercolith, coprolith, appendicolith or concretion) is a fecal concretion or pellet occupying the appendiceal lumen and is thought to be the result of a low fiber diet that accompanies a Western society lifestyle [11]. Popularized by early findings of giant surgeons [2] and propagated by animal studies showing appendicitis after appendiceal obstruction in rabbits [12], current surgical textbooks support the role of a fecalith as a major cause of appendicitis in adults [1].

The present analysis was stimulated by an unusual case in which a patient presented with a history of abdominal pain and had a large fecalith within the entire lumen of the appendix but did not cause appendicitis. A review of 257 veteran patients who had undergone an appendectomy for acute appendicitis at our institution, only 15.6% had fecaliths on the pathological specimen. A review of the literature showed that less than a quarter of patients presenting with appendicitis demonstrated a fecalith either radiographically, in the operating room, or on final pathology examination (Table 1).

The present case negates the role of the fecalith in the infectious process of appendicitis, even if it is very large. Our case series consists of veteran patients presenting with acute appendicitis and shows that fecaliths are found rarely in this patient population. The literature review demonstrated the following findings: (i) There is a wide range in the incidence of fecaliths leading to appendicitis; (ii) more cases of a normal appendix have been documented that have incidental fecaliths within the lumen compared to the frequency of a fecalith causing appendicitis; (iii) A temporal variation in the incidence of fecaliths as a cause of acute appendicitis has been proposed by Sigh *et al.* that divides this era prior to the 1970s and after that to current studies [4] and (iv) overall, there is an association between fecaliths and a risk of perforated appendicitis.

A fecalith is, therefore, an infrequent cause of appendicitis as suggested by textbooks. Other causes of appendicitis have been proposed and reviewed by Norman J Carr [13] and include appendiceal ulceration leading to infection, hygiene (enteric infection as a result of exposure of environmental factors), diet (appendicitis is less common in developing countries), ischemia, trauma, genetics, foreign bodies and type I hypersensitivity, and increased contractability of the appendix [13].

Thus, the current literature argues against a fecalith leading to acute appendicitis, but when present it is more likely to lead to perforation. This should be considered when assessing a patient for NOM of early appendicitis.

## References

[1] Cameron JL, Cameron AM Current Surgial Therapy. 10th edn. Philadelphia: Mosby, 2011:219.

[2] Fitz RH Perforating inflammation of the vermiform appendix. Am J Med Sci 1886;92:321–46.

[3] Arnbjornsson E, Bengmark S Obstruction of the appendix lumen in relation to pathogenesis of acute appendicitis. Acta Chir Scand 1983;149:789–91. 6666496

[4] Singh JP, Mariadason JG Role of the faecolith in modern-day appendicitis. Ann R Coll Surg Engl 2013;95:48–51. 23317728 10.1308/003588413X13511609954851 PMC3964638

[5] Khan MS, Chaudhry MBH, Shahzad N, Tariq M, Memon WA, Alvi AR Risk of appendicitis in patients with incidentally discovered appendicoliths. J Surg Res 2018;221:84–87. 29229158 10.1016/j.jss.2017.08.021

[6] Maenza RL, Smith L, Wolfson AB The myth of the fecalith. Am J Emerg Med 1996;14:394–97. 8768164 10.1016/S0735-6757(96)90058-3

[7] Shindoh J, Niwa H, Kawai K et al. Predictive factors for negative outcomes in initial non-operative management of suspected appendicitis. J Gastrointest Surg 2010;14:309–14. 19936849 10.1007/s11605-009-1094-1

[8] Renteria O, Shahid Z, Huerta S Outcomes of appendectomy in elderly veteran patients. Surgery 2018;164:460–65. 29914654 10.1016/j.surg.2018.04.027

[9] Jones BA, Demetriades D, Segal I, Burkitt DP The prevalence of appendiceal fecaliths in patients with and without appendicitis. A comparative study from Canada and South Africa. Ann Surg 1985;202:80–82. 2990360 10.1097/00000658-198507000-00013 PMC1250841

[10] Pieper R, Kager L, Tidefeldt U Obstruction of appendix vermiformis causing acute appendicitis. An experimental study in the rabbit. Acta Chir Scand 1982;148:63–72. 7136413

[11] Carr NJ The pathology of acute appendicitis. Ann Diagn Pathol 2000;4:46–58. 10684382 10.1016/s1092-9134(00)90011-x

[12] Bowers WF Appendicitis with especial reference to pathogenesis, bacteriology and healing. Arch Surg 1939;39:362–22.

[13] Steinert R, Hareide I, Christiansen T Roentgenologic examination of acute appendicitis. Acta Radiol 1943;24:13–37.

[14] Collins DC 71,000 human appendix specimens. A final report, summarizing forty Years' study. Am J Proctol 1963;14:265–81. 14098730

[15] Shaw RE Appendix calculi and acute appendicitis. Br J Surg 1965;52:451–59. 14296777 10.1002/bjs.1800520613

[16] Burkitt DP, Moolgaokar AS, Tovey FI Aetiology of appendicitis. Br Med J 1979;1:620. 10.1136/bmj.1.6163.620 PMC1598350 427491

[17] Butler C Surgical pathology of acute appendicitis. Hum Pathol 1981;12:870–78. 7298048 10.1016/s0046-8177(81)80190-6

[18] Chang AR An analysis of the pathology of 3003 appendices. Aust N Z J Surg 1981;51:169–78. 6940546 10.1111/j.1445-2197.1981.tb05932.x

[19] Andreou P, Blain S, Du Boulay CE A histopathological study of the appendix at autopsy and after surgical resection. Histopathology 1990;17:427–31. 2076868 10.1111/j.1365-2559.1990.tb00763.x

[20] Babekir AR, Devi N Analysis of the pathology of 405 appendices. East Afr Med J 1990;67:599–602. 2253567

[21] Herscu G, Kong A, Russell D et al. Retrocecal appendix location and perforation at presentation. Am Surg 2006;72:890–93. 17058728

[22] Marudanayagam R, Williams GT, Rees BI Review of the pathological results of 2660 appendicectomy specimens. J Gastroenterol 2006;41:745–49. 16988762 10.1007/s00535-006-1855-5

[23] Sgourakis G, Sotiropoulos GC, Molmenti EP et al. Are acute exacerbations of chronic inflammatory appendicitis triggered by coprostasis and/or coproliths? World J Gastroenterol 2008;14:3179–82. 18506922 10.3748/wjg.14.3179 PMC2712849

[24] Makaju R, Mohammad A, Shakya A Acute appendicitis: analysis of 518 histopathologically diagnosed cases at the Kathmandu University Hospital, Nepal. Kathmandu Univ Med J (KUMJ) 2010;8:227–30. 21209541 10.3126/kumj.v8i2.3564

[25] Engin O, Muratli A, Ucar AD, Tekin V, Calik B, Tosun A The importance of fecaliths in the aetiology of acute appendicitis. Chirurgia (Bucur) 2012;107:756–60. 23294954

[26] Chandrasegaram MD, Rothwell LA, An EI, Miller RJ Pathologies of the appendix: a 10-year review of 4670 appendicectomy specimens. ANZ J Surg 2012;82:844–47. 22924871 10.1111/j.1445-2197.2012.06185.x

[27] Ramdass MJ, Young Sing Q, Milne D, Mooteeram J, Barrow S Association between the appendix and the fecalith in adults. Can J Surg 2015;58:10–14. 25427333 10.1503/cjs.002014 PMC4309758

[28] Kim MS, Park HW, Park JY et al. Differentiation of early perforated from nonperforated appendicitis: MDCT findings, MDCT diagnostic performance, and clinical outcome. Abdom Imaging 2014;39:459–66. 24633221 10.1007/s00261-014-0117-x

[29] Iqbal Z, Uddin MT, Gulsharif F, Ahmad ZS, Jan Y, Azisulah F Determination of frequency of fecolith in perforated appendicitis. Ophthalmology Update 2015;13:308–10.

[30] Westfall KM, Charles AG Risk of perforation in the era of nonemergent management for acute appendicitis. Am Surg 2019;85:1209–12. 31775960 10.1177/000313481908501124

[31] Kulvatunyou N, Zimmerman SA, Joseph B et al. Risk factors for perforated appendicitis in the acute care surgery era-minimizing the Patient's delayed presentation factor. J Surg Res 2019;238:113–18. 30769247 10.1016/j.jss.2019.01.031

[32] Moskowitz E, Khan AD, Cribari C, Schroeppel TJ Size matters: Computed tomographic measurements of the appendix in emergency department scans. Am J Surg 2019;218:271–74. 30558802 10.1016/j.amjsurg.2018.12.010

